# Lung cancer mortality and associated predictors: systematic review using 32 scientific research findings

**DOI:** 10.3389/fonc.2023.1308897

**Published:** 2023-12-14

**Authors:** Lijalem Melie Tesfaw, Zelalem G. Dessie, Haile Mekonnen Fenta

**Affiliations:** ^1^ Departement of Statistics, Bahir Dar University, Bahir Dar, Ethiopia; ^2^ Epidemiology and Biostatistics Division, School of Public Health, Queensland University, Brisbane, QLD, Australia; ^3^ School of Mathematics, Statistics and Computer Science, University of KwaZulu-Natal, Durban, South Africa

**Keywords:** lung cancer, mortality, predictors, systematic review, global

## Abstract

**Background:**

Cancer is a chronic disease brought on by mutations to the genes that control our cells’ functions and become the most common cause of mortality and comorbidities. Thus, this study aimed to assess the comprehensive and common mortality-related risk factors of lung cancer using more than thirty scientific research papers.

**Methods:**

Possible risk factors contributing to lung cancer mortality were assessed across 201 studies sourced from electronic databases, including Google Scholar, Cochrane Library, Web of Science (WOS), EMBASE, Medline/PubMed, the Lung Cancer Open Research Dataset Challenge, and Scopus. Out of these, 32 studies meeting the eligibility criteria for meta-analysis were included. Due to the heterogeneous nature of the studies, a random-effects model was applied to estimate the pooled effects of covariates.

**Results:**

The overall prevalence of mortality rate was 10% with a 95% confidence interval of 6 and 16%. Twenty studies (62.50%) studies included in this study considered the ages of lung cancer patients as the risk factors for mortality. Whereas, eighteen (56.25%) and thirteen (40.63%) studies incorporated the gender and smoking status of patients respectively. The comorbidities of lung cancer mortality such as cardiovascular disease, hypertension, diabetes, and pneumonia were also involved in 7 (21.90%), 6 (18.75%), 5 (15.63%), and 2 (6.25%) studies, respectively. Patients of older age are more likely to die as compared to patients of younger age. Similarly, lung patients who had smoking practice were more likely to die as compared to patients who hadn’t practiced smoking

**Conclusion:**

The mortality rate of lung cancer patients is considerably high. Older age, gender, stage, and comorbidities such as cardiovascular, hypertension, and diabetes have a significant positive effect on lung cancer mortality. The study results will contribute to future research, management, and prevention strategies for lung cancer.

## Introduction

Cancer is a chronic disease brought on by mutations to the genes that control our cells’ functions, particularly their growth and division, which results in uncontrolled cell growth and division that forms malignant tumors and spreads to nearby organs (1). In 2015, the Global Burden of Disease Cancer study found that, with 8 million deaths, cancer was the second greatest cause of death worldwide, with cardiovascular illnesses taking the top spot (2).

According to 2018 World Cancer statistics, there were an estimated 18 million cancer cases around the world, of which 9.5 million cases were males and 8.5 million in females (3). Recently National Cancer Institute showed that there are more than 100 types of cancer which are usually named for the organs or tissues where the cancers form (2, 4). With 2,093,876 cases or 12.3% of the total, lung cancer is the first and most often diagnosed cancer worldwide. Among the top five most frequently diagnosed cancers, breast (2,088,849 cases, 12.2% of the total), colorectal (1,800,977 cases, 10% of the total), prostate (1,276,106 cases, 9.8% of the total), and stomach (1,033,701 cases, 5% of the total) are ranked second, third, fourth, and fifth, respectively (2). As a result, this study was focused on a meta-analysis of mortality-related risk factors of lung cancer.

Lung cancer is a malignancy that typically develops in the cells lining the airways of the lung. It remained the leading cause of cancer death, with an estimated 1.8 million deaths (18%), followed by colorectal (9.4%), liver (8.3%), stomach (7.7%), and female breast (6.9%) cancers (4, 5). Lung cancer has already become a threat to public health around the world with nearly 2 million cases and deaths in 2020. The cases of lung cancer annually are anticipated to reach 3.8 million in 2050, even with current risk levels and age-specific rates (6). Several studies reported that socioeconomic, demographic, biological, and behavioral factors are important determinants of lung cancer mortality (5). Among these age (7–10), sex (11–14), cigarette smoking (8, 9, 12, 13), stage (10, 14–16), infectious lung disease (ILD) (7, 17, 18), body mass index (BMI) (12, 13, 19, 20), diabetes (13, 15, 19, 21), and hypertension (12, 17, 19) are the most common factors associated with lung cancer mortality.

In regards to gender, following prostate and colorectal cancer, lung cancer is the most common cancer and the main cause of cancer death in men. The lung cancer death rates among women whose husbands had ever smoked during the current marriage were 20% higher than those among those who were married to never-smokers. Cigarette smoking is the most important preventable risk factor for lung cancer, which is the leading cause of cancer mortality (6). Lung cancer risk is higher in people with low BMI. When the analysis was limited to lifetime nonsmokers, an increased risk of lung cancer associated with a family history of the disease was found, though this did not reach statistical significance (11, 22).

Despite, the lung cancer mortality rates are increasing and numerous studies conducted (8, 17, 18, 21, 23, 24) to identify the potential risk factors, still a lack of studies that show common causes of mortality due to lung cancer. Several studies were done on lung cancer to identify associated factors of it. However, they are limited to some specific locations, cases, and attributes. Thus, this study aimed to assess the comprehensive and common mortality-related risk factors of lung cancer using more than thirty scientific research papers. In this study, we provide a comprehensive and comparable investigation of mortality-related risk factors of lung cancer global level.

## Materials and methods

### Study protocol

To evaluate the association between lung cancer mortality and comorbidities, as well as other socioeconomic, demographic, and biological factors. The study implemented and followed PRISMA procedures to execute the meta-analysis of the articles identified through our systematic reviews.

### Search strategy

We systematically searched electronic databases up until 10 December 2022, including Google Scholar, Cochrane Library, Web of Sciences (WOS), EMBASE, Medline/PubMed, lung cancer Open Research Dataset Challenge, and Scopus. The search strategy was as follows: (lung cancer mortality OR lung neoplasms mortality OR cancer death rate) AND (risk factors OR predictors OR determinants) AND (adult patients OR lung cancer patients OR lung cancer survivors). The search was also narrowed down to articles that examined laboratory data, pre-existing comorbidities, clinical status, and demographic traits as potential indicators of lung cancer’s fatal outcome. The time and language of publications were not subject to any limitations. We downloaded the literature results into EndNote X9 to speed up the screening procedure.

### Eligibility criteria

**Figure 1:**
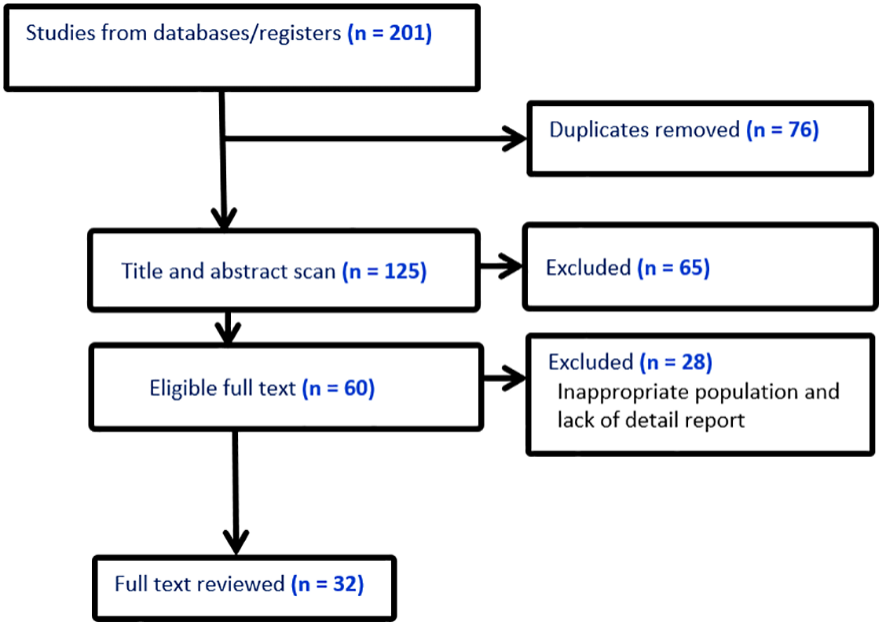
Literature screening flow chart.

After duplicates were eliminated, the initial search results were checked for relevance by both authors using titles and abstracts. The eligibility requirements were examined in the complete texts (**Figure 1**). Excluded from the analysis were studies without an abstract or full text, correspondence, studies on infants only, editorials, reviews, qualitative studies, books, theses, expert opinion papers, and review articles. We also used studies that only provided odds ratios (ORs), hazard ratios (HRs), or relative risks (RR) along with 95% confidence intervals (CI) for the association between demographic, epidemiological, or clinical characteristics and fatal outcomes of lung cancer among the eligible studies. The overall studies included in this study was presented in **Table 1**.

**Table 1:** Characteristics of studies included in the systematic review and meta-analysis on lung cancer mortality.

Authors (year)	Country	Sample size	Death	RR/OR or HR (95%CI)
Zhang, B., et al. (8)	Canada	49,165	106	Smoking: Yes: HR=14.04 (7.56-26.07) Age: Old (>50):HR=1.74(1.45-1.87)
Chiyo, M. et al. (7)	Japan	931	293	Gender: male: HR =1.766 (1.304-2.401) Age: HR=1.015 (1.008-1.027) ILD: Yes: HR=1.903(1.012-3.236)
Frostad, A. (23)	Norway	19,998	6710	Age: Old: aOR=1.04 (1.01-1.06)
Kabat, G.C., et al. (25)	China	1590	131	Duration of HRT use: Yes: aHR=1.51 (1.14–1.99). Contraceptive use: Yes: HR=0.91 (0.78–1.06)
Yang, L., et al. (11)	China	225, 721	2145	Follow up: higher: HR=0.75(0.63-0.93) Residence: Urban: HR=2.28 (2.06-2.52) Sex: Male: HR=1.57 (1.47-1.67) Smoking: Yes: HR=1.77 (1.67-1.87)
Roth, K., et al. (10)	Norway	148	40	Age: >=70=HR= 1.93 (1.14, 3.28) Stage: HR=Stage IB= 1.63 (0.92, 2.89) HR=Stage II-IV=4.16 (1.92, 9.05)
Chae, K.J et al. (9)	Korea	612	220	Age: in yr=HR= 1.03 (1.01–1.05) Smoke: yes=HR= 1.68 (1.22–2.32) History: yes=HR= 1.44 (1.00–2.06)
Kuderer, N.M (12).	Canada	1998	472	Age: a year=HR=1.013 (1.003–1.023) Sex: male=HR=1.183 (0.979–1.430) BMI: <35 kg/m2=HR=0.968 (0.611–1.531) Smoke: Yes=HR=1.551 (1.185–2.030) HPT: Yes=HR=1.131 (0.937–1.365) Anaemia: Yes=HR=1.471 (1.185–1.826)
Duran, A.O (13)	Turkey	330	125	Age: year=1.008 (0.990-1.027) Sex: Male=0.863 (0.479-1.554) BMI: 0.967 (0.924-1.012) Smoking: 1.518 (0.890-2.589) Diabetes: 1.200 (0.655-2.199) HPT: Yes=1.076 (0.627-1.847) Cardiac disease: Yes=1.330 (0.656-2.695)
Adair, T (22).	Australia	2480	249	Tobacco: Yes: OR=1.066 (1.002-1.090)
Matakidou, A., et al. (26)	UK	2561	191	Smoker: Yes: RR= 7.15 (5.70–8.96) Age: 60+: RR=2.02 (1.22–3.34) Relatives: Yes: RR=1.90 (1.30–4.25)
Stukenborg, G.J (17).	USA	14,456	519	Liver disease: Yes=RR=2.60 (1.16-5.87) Pneumonia: Yes=RR= 1.73 (1.24-2.42) Hypertension: Yes=RR=1.83 (1.19-2.81)
Licker, M.J., et al. (27)	Switzerland	1222	36	Respiratory complication: Yes=RR=1.9 (1.9-6.6)
Lauritsen, J.M (28).	Danish	439	60	Smoking=Yes=2.02 (0.80, 6.78)
Powell, H.A. et al. (14)	UK	10991	981	Sex=Male=OR= 1.62(1.17 to 2.25) Age=85+=OR= 2.35(1.12 to 9.01) Stage=IIIA/IA= 2.18 (1.25 to 3.81)
Kirkland, R.S (18).	USA	882	67	interstitial lung disease=Yes=OR=6.14(1.9-19.4) pulmonary HT=Yes=OR=3.1(1.6-6.2) diabetes mellitus=Yes=OR=2.0(1.1-3.3)
Thomas, P.A (29).	France	4498	351	Age: >65=OR=2.1(1.5-2.9) BMI: Underweight: OR=2.2(1.2-4.0) Overweight: OR=0.60(0.4-0.9)
Rosen, J.E (30).	USA	119,146	4051	Age: 5-yr increase=OR=1.5(1.25, 1.77) Sex: Male=OR=1.8 (1.3, 3.28) Race: Non-white=OR=1.10(0.8, 1.56)
Gibson, A.J (31).	Canada	1044	233	Sex: Male=OR=1.48 (1.12–1.95) never-smokers=OR=0.62 (0.41–0.95) Advanced disease at diagnosis=OR=1.85 (1.19–2.88) Receiving systemic anti-cancer therapies (SACT)=OR=0.65 (0.49–0.86)
Kozower, B.D (19)	USA	18,800	413	Age: 10-year increase=OR=1.84 (1.58, 2.15) Sex: Male=OR=1.36 (1.07, 1.73) BMI: 10KG/M2 increase=OR=0.74 (0.58, 0.94) HPT: Yes:=OR=1.10 (0.85, 1.43) Diabetes: Yes:=OR=1.15 (0.82, 1.59) Cardiovascular: Yes=OR=1.09 (0.78, 1.51) Dialysis: Yes=OR=3.97 (1.48,10.64)
Jean, R.A (32)	USA/Vreginia	6435	120	Age: year=OR=1.03(1.013-1.056) Sex: male=OR=1.83 (1.199-2.789) DM: Yes=OR=2.57(1.379-4.808)
Romano, P.S (21).	USA/California	12,439	441	Sex: male=OR=1.5 (1.2,1.8) Age: >79=OR=5.4 (3.5,8.4) Heart: Yes=OR=1.8 (1.5,2.2) Diabetes: Yes=OR=1.5 (1.1,2.2)
Kates, M., et al. (15)	USA/NewYork	1844	655	Sex: male=OR= 1.45 (1.22–1.72) Age: 80-80=OR=2.09 (1.60–2.75) Stage: III vs I=OR=1.27 (1.02–1.59) Heart Failure: Yes=OR=1.54 (1.24–1.93) Cardiovascular: Yes=OR=1.75 (1.39–2.22) Diabetes: Yes=OR=1.70 (1.18–2.45)
Breslow, R.A (33).	USA	20195	158	Smoking: Yes= RR=1.2 (0.8-1.8)
Bessö, A., et al. (34)	Sweden	316	92	Sex: male=RR=1.3(0.76–2.62) Smoking: Yes=3.32 (1.35–8.20)
Borsoi, L (35).	Austria	1000000	503/10-6	Sex: Female=RR= 1.12 (0.81-1.66) Age: RR=0.77(0.29-1.22)
Cardenas, V.M (36).	USA	508,576	5,469	Smoke: Yes=RR= 1.2(0.8-1.8) Sex: Female=RR=1.45 (0.74-2.55)
Chow, W.H., et al. (37)	USA	5263	219	Smoking: Yes=RR=3.5 (1.0-12.6) Beer: Yes=RR=1.8 (1.10-3.30) Meat: Yes=RR=1.3(0.7-2.3) Vitamin C: high=RR=0.8(0.5-1.2)
Stoelben, E.,et al. (16)	Germany	1281	131	Sex: Female=RR=0.56(0.30–1.06) Age: >75=RR=2.46 (1.17–5.16) Stage: I vs II 5.58 1.83–17.07 I vs IIIa 9.01 3.52–23.04 I vs IIIb 17.41 6.62–45.80 I vs IV 21.81 8.29–57.38
Sokolnikov, M.E (38).	Russia	17,740	681	Sex: Male: RR=7.1 (4.9–10) Age: 75+=RR=4.1 (0.9–10)
Kuehnl, A (20).	Germany	31	22	Sex: male=RR=2.08 0.478–9.025 Age: <65 vs>65=RR=0.98 0.934–1.035 BMI: <25 vs >25= RR=0.95 0.856–1.057 Grade: G1/G2 vs G3/G4=RR=0.32 0.110–0.909
Mansfield, A.S, et al. (24)	USA	759	568	Surgery= HR, 0.35; 95% CI, 0.18–0.68 Chemotherapy=HR, 2.2; 95% CI, 1.2–4.3 Radiation (ref.) KRS (yes vs no)=HR, 1.7; 95% CI, 1.4–2.2 Age: >76= HR, 1.45: 95% CI, 1.12-2.66 Sex: male=HR, 1.32: 95% CI, 1.06-3.10

### Data extraction and assessment for study quality

The downloaded EndNote X9 search outputs were independently reviewed for inclusion by each author. Discussion and consensus among the authors were used to settle any differences. The first author’s name, country, assessment techniques, sample size, study design, publication year, demographic and clinical variables (such as gender, age, and comorbidities), outcome (mortality), exposure (risk factors), and adjusted odds ratios or hazard ratios or relative risks were all extracted by all authors.

Using the Newcastle-Ottawa method, the authors independently assessed the articles’ quality methodological approach. This method relied on three main elements to evaluate the quality of the papers: evaluation of the results, comparability of the study groups, and patient selection methods. The seven domains in the Newcastle-Ottawa technique were scored from 3 to 0 (i.e., from low to high bias), and their average score was taken.

### Statistical analysis

To determine the relationship between risk factors and the likelihood that lung cancer will be fatal, we used ORs, RRs, or HRs (and their 95%CI) that were peer-reviewed and published. The expected between-study heterogeneity has been taken into consideration when computing a mixed-effect model. Cochran’s Q test was used to determine whether there was heterogeneity in effect sizes; a significant Q value suggests that there is heterogeneity rather than homogeneity. The *I* ^2^ statistic was used to calculate the percentage of the total variance that could be attributed to study heterogeneity (39). The *I* ^2^ values between 60% and 90%, 40% and 59%, and 0% and 39% were regarded as severe, moderate, and mild, respectively. For evaluating publication bias, funnel plots with an Egger-weighted regression test were used (40). The pooled odds ratio, relative risk, and hazard ratio were calculated and publication bias was examined using STATA version 17 and R-4.0.2 statistical software, respectively.

## Results

In this study, a total of 201 publications on the mortality of lung cancer were identified using so many sites such as Google Scholar, Cochrane Library, Web of Sciences (WOS), EMBASE, Medline/PubMed, cancer research database (WHO), lung cancer open research dataset challenge, and Scopus database, of which, 15 studies that did not have numbers of hospital death, 35 reviews, 15 non-English, and 76 duplicates were excluded. Among the remaining 60 studies, 28 did not report cross-tabulation with ORs or HRs, or RRs. Consequently, we got only 32 studies that satisfied all the eligibility criteria (see **Figure 1**). The studies considered in this meta-analysis consists of numerous mortality-related risk factor of lung cancer diseases (6, 8, 10, 11, 16, 18, 25, 26, 29, 30, 32) (see **Table 1**). The effect of each risk factor on mortality of lung cancer was measured and estimated using adjusted odds ratio (OR), relative risk (RR), or hazard ratio (HR).

**Figure 2:**
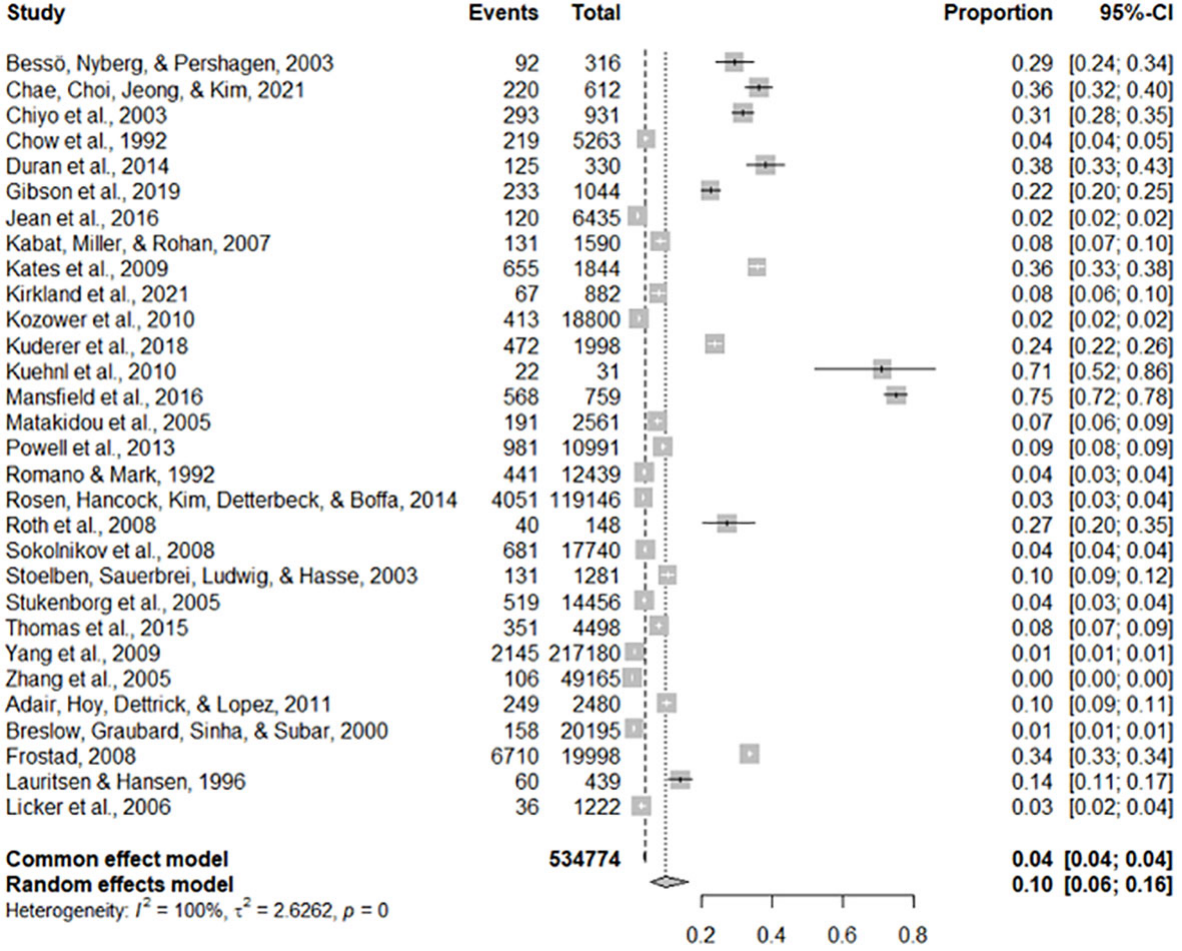
The overall mortality rate of lung cancer.

**Figure 3:**
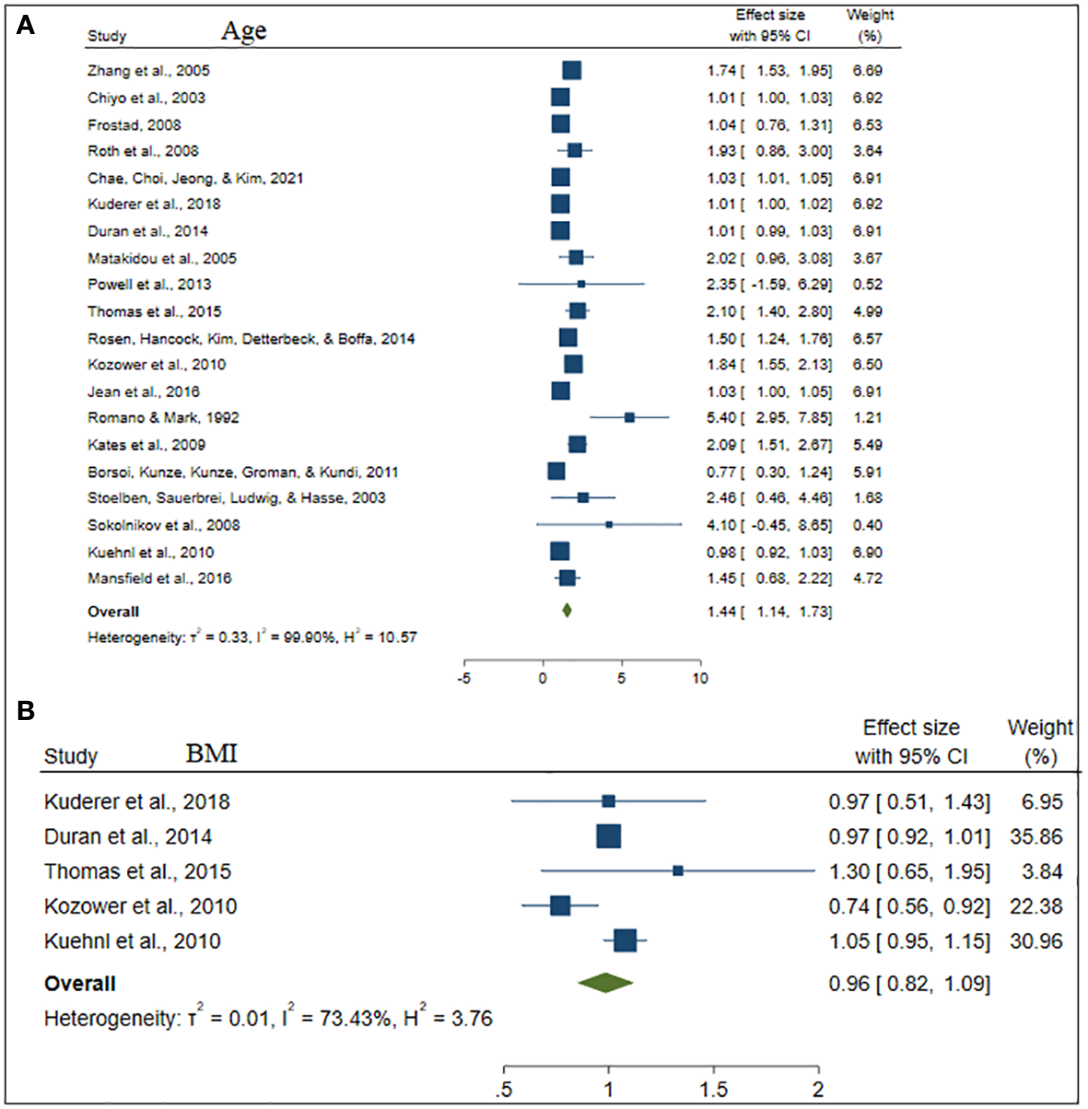
The forest plot of the effect size age **(A)** and BMI **(B)**.

**Figure 4:**
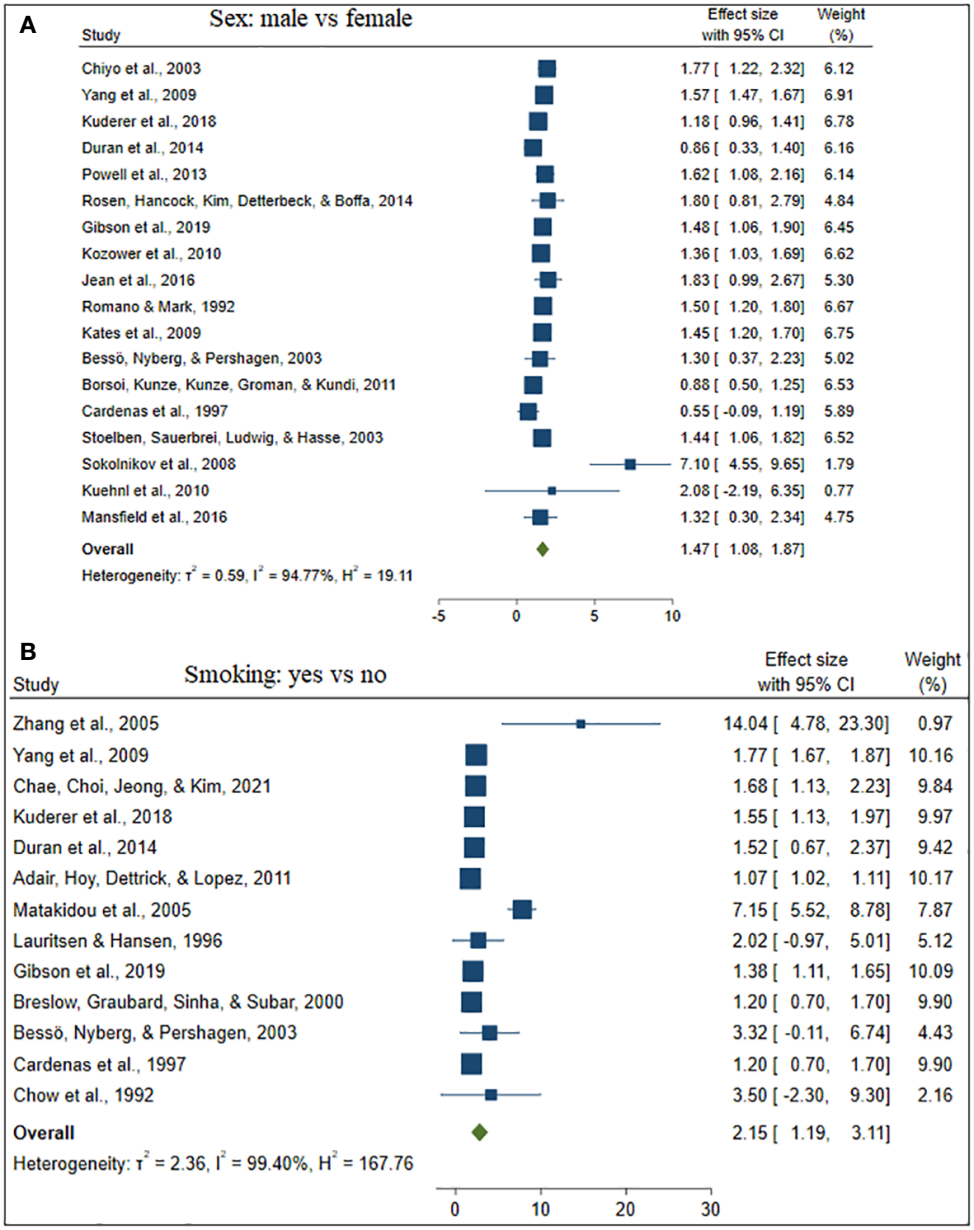
The forest plot of the effect size sex **(A)** and smoking **(B)**.

The estimate of each risk factor was the pooled of OR, RR, and HR. This was done using a forest plot in **Figures 1**–**4** for risk factors age, BMI; sex, smoking; cardiovascular, stage; hypertension, diabetes; and Interstitial Lung Disease (ILD), Pneumonia, respectively. Out of the 32 studies, ten, fourteen, and eight studies provided the effect of risk factors were estimated using HR, OR, and RR respectively. The Meta-analysis included studies with retrospective and prospective study designs. The mortality rate noticed in retrospective studies was lower as compared to prospective studies. For instance, in the retrospective study conducted by Zhang et al. the mortality rate was 106 to 49165 which is almost null, whereas, in the prospective study conducted by Mansfield et al, the mortality rate was 568 to 759 which is almost 75 percent of the total. The overall prevalence of mortality rate was 10% with a 95% confidence interval of 6 and 16% (see **Figure 2**).

Twenty studies (62.50%) studies included in this study considered the ages of lung cancer patients as the risk factors for mortality. Whereas, eighteen (56.25%) and thirteen (40.63%) studies were incorporate the gender and smoking status of patients respectively. The comorbidities of lung cancer mortality such as cardiovascular disease, hypertension, diabetes, and pneumonia were also involved in 7 (21.90%), 6 (18.75%), 5 (15.63%), and 2 (6.25%) studies, respectively (see **Table 2**).

**Table 2:** Results of meta-analysis based on demographic and clinical variables associated with lung cancer mortality.

Risk factors	Numbers of studies (%)	Effect size (95% CI)	Heterogeneity	
			I^2^	P-value
Older age	20 (62.50)	2.61(1.75-3.47)	99.97	0.000
BMI	5 (15.63)	0.96(0.82-1.09)	73.43	0.064
Gender: Male vs Female	18 (56.25)	1.47(1.08-1.87)	94.77	0.000
Smoking status: Yes vs No	13 (40.63)	2.15(1.19-3.11)	99.40	0.000
Cardiovascular: Yes vs No	7 (21.90)	1.57(1.28-1.86)	48.34	0.000
Stage: Yes vs No	6 (18.75)	1.60(1.21-1.99)	32.55	0.020
Hypertension: Yes vs No	6 (18.75)	1.19(1.04-1.34)	0.00	0.000
Diabetes: Yes vs No	5 (15.63)	1.43(1.14-1.72)	15.23	0.000
ILD	2 (6.25)	1.97(0.86-3.08)	0.00	0.000
Pneumonia	2 (6.25)	1.80(1.23-2.37)	0.00	0.000

**Figure 5:**
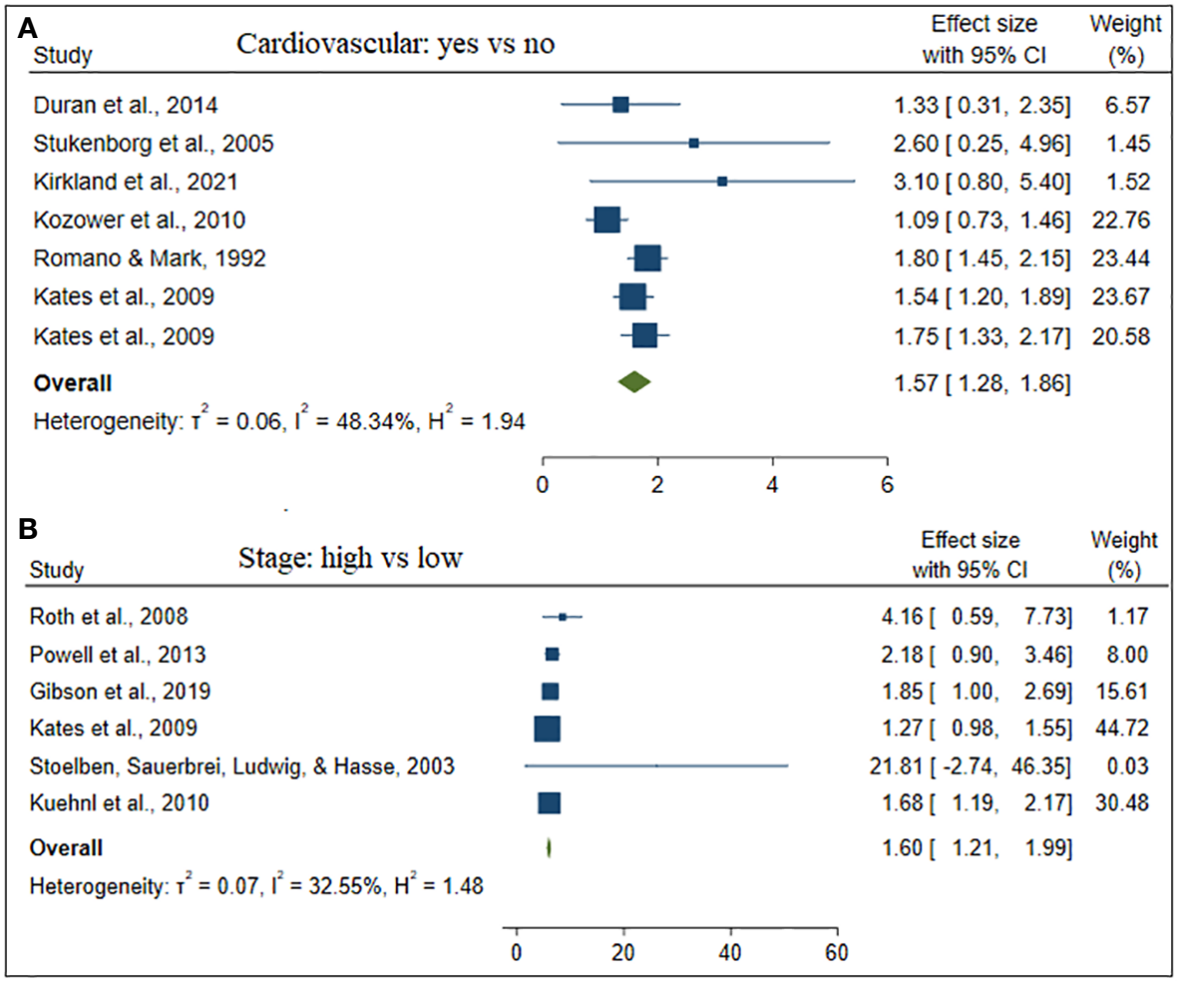
The forest plot of the effect size cardiovascular **(A)** and stage **(B)**.

**Figure 6:**
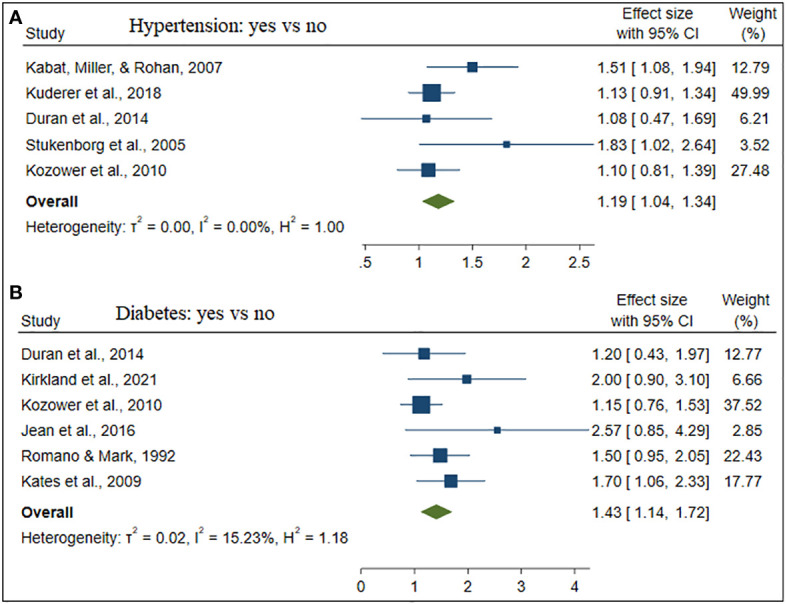
The forest plot of the effect size hypertension **(A)** and diabetes **(B)**.

Besides, **Table 2** depicted the overall effect size demographic and clinical variables associated with lung cancer mortality. Except for the BMI of the patient determinants such as patient age, gender, smoking, cardiovascular, stage of cancer metastasis, diabetes and pneumonia has a significant positive effect on lung cancer mortality. For instance, patients of older age are more likely to die as compared to patients of younger age. Similarly, lung patients who had smoking practice were more likely to die as compared to patients who hadn’t practiced smoking. The estimated effects of covariates for each study separately and aggregate/overall estimated effect were also presented using a forest plot in **Figures 3**–**6**.

**Figure 7:**
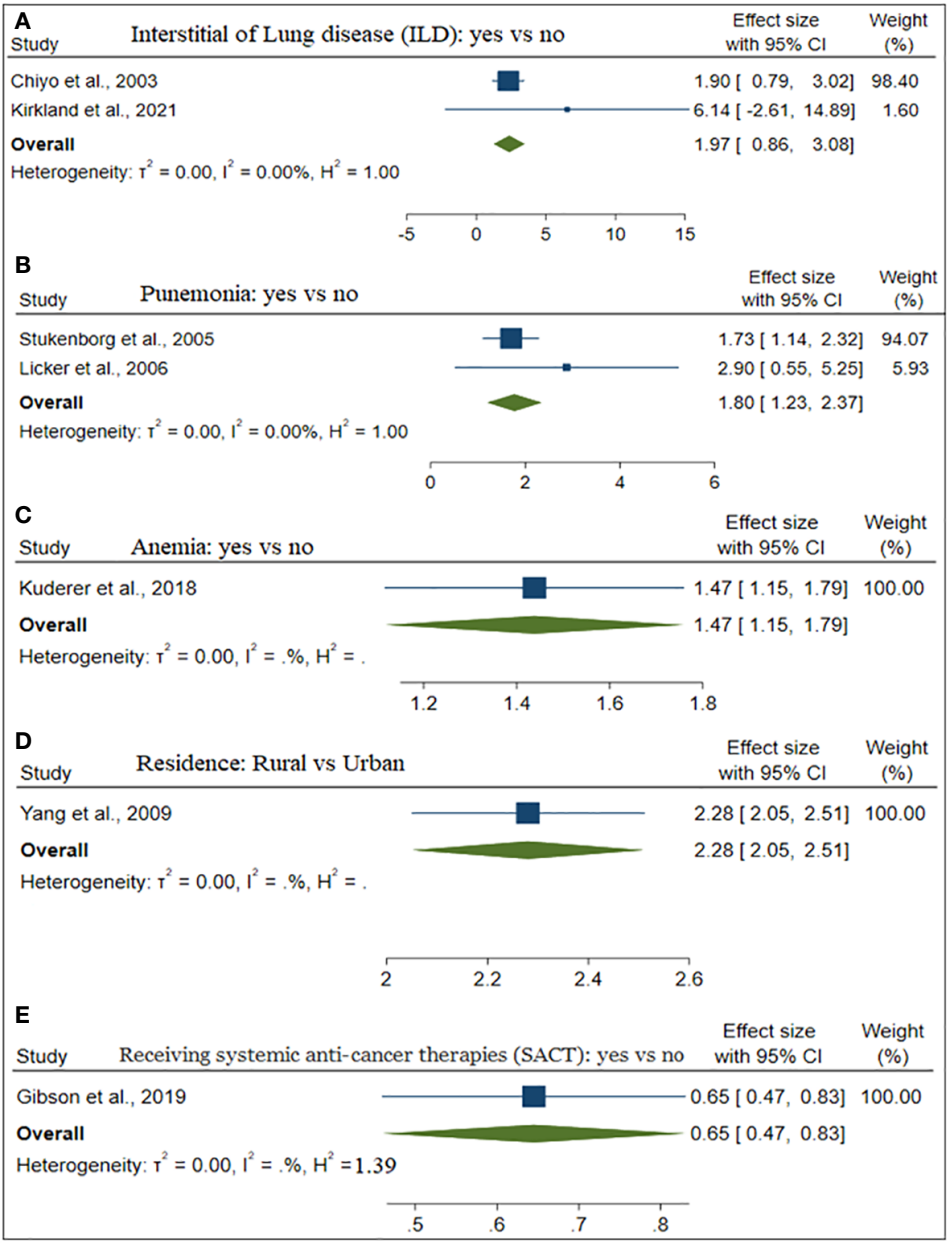
The forest plot of the effect size ILD **(A)**, Pneumonia **(B)**, Anemia **(C)**, and Residence **(D)**.

Despite this, only one single study on the effect of anemia, residence, and receiving systematic anti-cancer treatment (SACT) on lung cancer mortality was included in this study (see **Figure 7**). Patients who had and lived in rural areas were more likely to die. In contrast, patients who took SACT were less likely to die.

**Figure 8:**
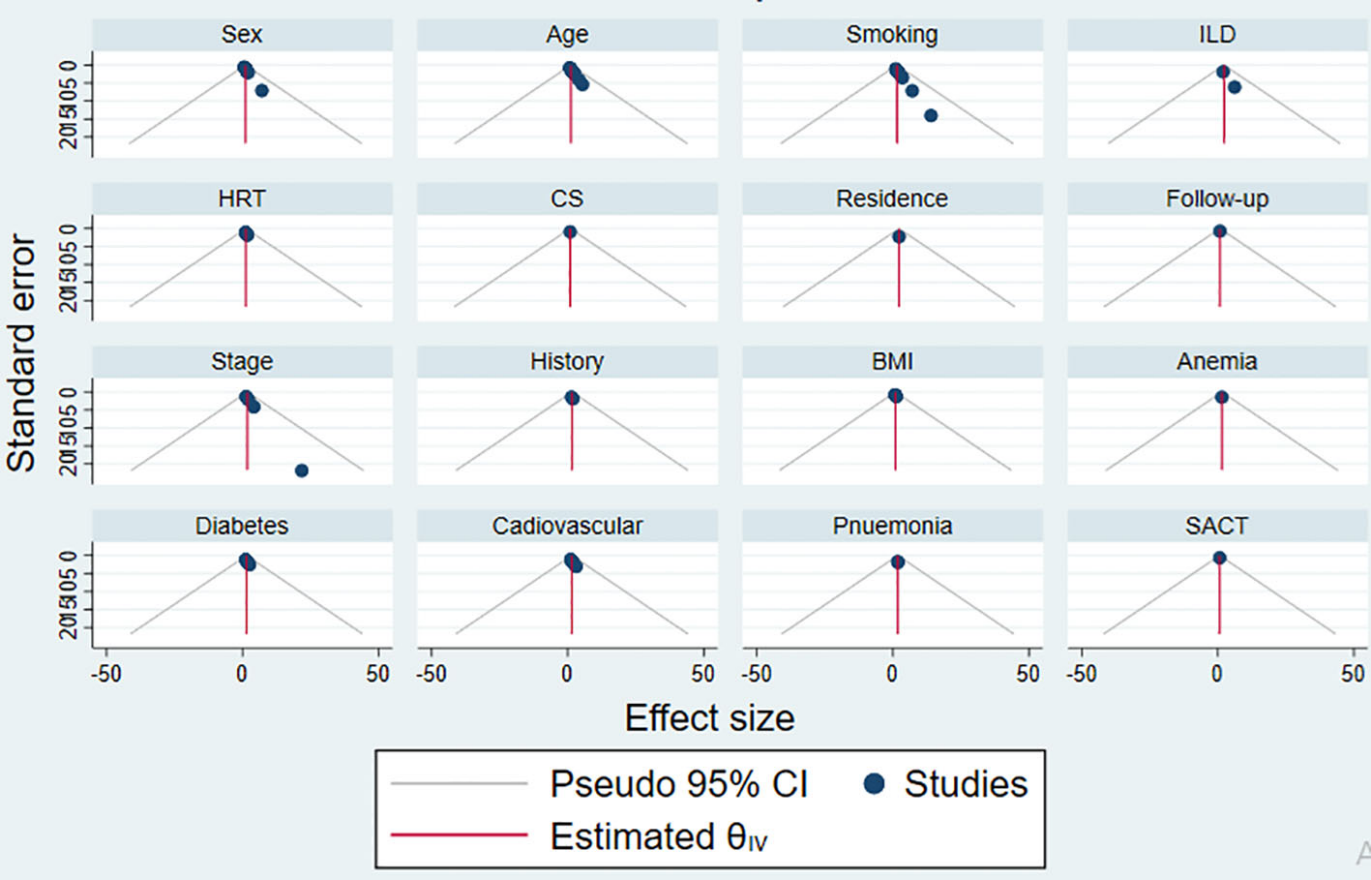
The funnel plot of each risk factors publication bias.

The goodness of the meta-analysis for each factor was considered using a funnel plot in **Figure 8**. The points within the funnel line indicate the systematic review analysis for the corresponding variable is a good fit.

## Discussion

This study presents a comprehensive systematic review examining the risk factors associated with lung cancer mortality. The objective was to comprehensively explore potential risk factors, including demographic, biological, behavioral, and socioeconomic determinants associated with lung cancer mortality, and to estimate the overall prevalence of lung cancer mortality. We delved into a total of 201 lung cancer studies sourced from diverse electronic databases, including Google Scholar, Cochrane Library, Web of Sciences (WOS), EMBASE, Medline/PubMed, the Lung Cancer Open Research Dataset Challenge, and Scopus. In the meantime, 32 studies that satisfy the eligibility criteria of the Meta-analysis were involved in this study. The majority of the studies consist of patients’ age, gender, and smoking status (7–15, 21, 24, 26, 29, 31).

Despite reports indicating a decline in the mortality rate of patients with lung cancer, it remains substantial. The overall mortality rate stands at 10%, signifying that, on average, ten out of every hundred lung cancer patients succumb to the disease. Put differently, there is a one in ten likelihood that a lung cancer patient will die. In 2012, an estimated 1.8 million new cases, accounting for 12.9% of the total, were recorded (41). In 2012, the regions with the highest lung cancer mortality rates per 100,000 were Central and Eastern Europe and Eastern Asia for males, and Northern America and Northern Europe for females. Conversely, the lowest rates were observed in sub-Saharan Africa for both males and females (42).

This study depicted that older age, gender, stage, and comorbidities such as cardiovascular, hypertension, and diabetes have a significant positive effect on lung cancer mortality. A study conducted on global trends of lung mortality revealed that the overall trend of lung cancer mortality among females is higher than among males (42), which contradicts the findings of this study states that males have a higher likelihood to be died as compared to females. The probability that an individual is affected by lung cancer increases as age does. This was in line with studies in (43, 44), which reported that lung cancer incidence and mortality rates steadily rise after the age of 30, reaching a peak between the ages of 75 and 79 for men and 70 to 74 for women. Due to less concomitant illness, younger individuals may be able to tolerate more rigorous multimodal therapy. Various factors, including higher prevalence of occupational hazards like asbestos exposure and common settings such as kitchens, where staff are exposed to smoke, may contribute to increased lung cancer risk among females in the population.

The majority of patients visit the hospital during the advanced stage (III/IV) of cancer, which leads to having an effective prescription or treatment. Thus, the stage of the cancer diseases affected the survival time of the patient (43). Communities who are living in rural areas had more vulnerability to death. This is common because of poor health facilities and leads unable to take treatment in time in rural areas as compared to the urban. If lung cancer is detected early and the right treatment is available, it may be curable. The place of residence of patients interrelates with lifestyle, which is a considerable risk factor for cancer (45). A potential obstacle to the effective management of future changes in incidence and mortality rate is the lack of or limited access to health care services in rural areas, particularly in developing nations.

The most frequent factor in both lung cancer incidence and mortality is smoking which is in line with studies (8, 46) state that lung cancer is directly caused by tobacco use, particularly cigarette smoking. Thus, an essential behavioral strategy for preventing lung cancer is quitting smoking. However, since this risk never goes back to normal, former smokers continue to have heightened risk compared to never smokers. Former smokers account for over 50% of instances of diagnosed lung cancer. About 58 percent of these occurrences happened in less developed areas, which is probably a reflection of the rising cigarette usage in these nations. Lung cancer is the most frequent kind of cancer death globally, accounting for 1.59 million projected deaths in 2012, even though exposure to tobacco smoke is avoidable (47, 48). In the last, this review synthesizes evidence from multiple studies, offering a more robust and reliable overview than individual studies. This helps in drawing more accurate conclusions. It provides a comprehensive understanding of the current state of knowledge regarding factors influencing lung cancer mortality. This is valuable for researchers, healthcare professionals, and policymakers.

Despite providing pooled estimates from 32 studies across 13 geographical locations, which may be seen as broadly representative of the pandemic, our systematic review has several limitations. Firstly, there is high heterogeneity, potentially due to substantial variations in sample sizes among studies and differences in study designs. Secondly, even with careful statistical adjustments, confounding variables can affect the validity of the results, as not all factors influencing lung cancer mortality are incorporated. Thirdly, it’s important to note that this study did not consider covariates related to treatment outcomes, survival rates, and disease progression in cancer patients. Lastly, some included studies had very small sample sizes, possibly limiting the identification of factors influencing lung cancer mortality.

## Conclusion

The mortality rate among lung cancer patients is notably elevated. Several factors contribute significantly to lung cancer mortality, including older age, gender, disease stage, and the presence of comorbidities such as cardiovascular conditions, hypertension, and diabetes. These findings hold substantial implications for the field of lung cancer research, management, and future prevention strategies. By shedding light on the influential factors behind lung cancer mortality, this study offers valuable insights that can inform more effective approaches to Comprehensive tobacco control policies, public awareness campaigns highlighting the dangers of smoking, and measures to improve air quality and regulate occupational exposures are essential. Access to healthcare services needs enhancement, focusing on early detection and treatment. Supporting smoking cessation efforts, promoting a healthy lifestyle, and integrating preventive measures into primary healthcare systems are vital components. International collaboration for knowledge sharing and resource allocation further strengthens the global fight against lung cancer combatting this deadly disease, ultimately leading to improved patient outcomes and reduced mortality rates.

## Data availability statement

The original contributions presented in the study are included in the article/supplementary material. Further inquiries can be directed to the corresponding author.

## Author contributions

LT: Conceptualization, Data curation, Formal Analysis, Investigation, Methodology, Software, Supervision, Validation, Visualization, Writing – original draft, Writing – review & editing. ZD: Data curation, Investigation, Methodology, Software, Supervision, Validation, Writing – review & editing. HF: Data curation, Formal Analysis, Investigation, Methodology, Software, Validation, Visualization, Writing – review & editing.
